# Knowledge, attitude, and practice of the 2009 Institute of Medicine (IOM) recommendations on the nutritional management of diabetes in pregnancy: an online national survey

**DOI:** 10.1007/s00592-022-01950-8

**Published:** 2022-09-02

**Authors:** Gloria Formoso, Cristina Bianchi, Silvia Burlina, Elisa Manicardi, Maria Angela Sculli, Veronica Resi, Laura Sciacca

**Affiliations:** 1 Interassociative Diabetes and Pregnancy Study Group, Italian Association of Diabetologists (AMD), Italian Society of Diabetology (SID), Rome, Italy; 2 Department of Medicine and Aging Sciences, Center for Advanced Studies and Technology (CAST, Ex CeSIMet) G. d’Annunzio University Chieti-Pescara, Chieti, Italy; 3 Metabolic Diseases and Diabetes Unit, University Hospital of Pisa, Pisa, Italy; 4 Department of Medicine, DIMED, University of Padova, Padova, Italy; 5 Diabetes Unit, Primary Health Care, Local Health Authority of Reggio Emilia-IRCCS, Reggio Emilia, Italy; 6 Endocrinology and Diabetes, Bianchi-Melacrino-Morelli Hospital, Reggio Calabria, Italy; 7 Endocrinology Unit, Fondazione IRCCS Ca’ Granda Ospedale Maggiore Policlinico, Milan, Italy; 8 Department of Clinical and Experimental Medicine, Endocrinology Section, University of Catania, Catania, Italy

**Keywords:** Gestational diabetes, Diet, Nutrition, Pregnancy, IOM guidelines, Weight gain

## Abstract

**Aims:**

As recommended by the Institute of Medicine (IOM), health practitioners should encourage a healthy nutrition and adequate weight gain during pregnancy in order to ensure favorable pregnancy and fetal outcomes, and to prevent diseases later in life for both mother and child. The purpose of this online survey was to determine the knowledge, attitude, and practice of the 2009 IOM recommendations among healthcare professionals managing nutritional therapy in pregnancies complicated by diabetes in Italy.

**Methods:**

A cross-sectional survey was conducted by using an online self-administered questionnaire undertaken between October and December 2021.

**Results:**

Of the 220 participants 89% were diabetologists/endocrinologists/internal medicine specialists and 11% dietitians/nutritionists. The survey found that the 53% of respondents provide a personalized diet to pregnant women with diabetes, while 32% a standard diet plan and only 15% healthy dietary advice. The 69% of the participants investigated for appropriate gestational weight gain, mainly based on pre-pregnancy BMI (96%), gestational weight gain (GWG) at first prenatal visit (80%) and presence of twin pregnancy (58%). Maternal weight gain was evaluated at each regularly scheduled prenatal visit and compared with IOM recommendations for the 87% of healthcare professionals. Diet plan was periodically re-evaluated and/or modified (90% of participants), based on inadequate maternal weight gain and/or fetal growth abnormalities (78%), trimester transition (53%), changes in physical activity and/or a “feel hungry” (50%).

**Conclusions:**

This survey reported the knowledge and attitude of IOM guidelines and the nutritional knowledge and practice of Italian professionals on the nutritional management of diabetes in pregnancy. The application of these recommendations seemed more feasible in clinics/team dedicated to "Diabetes in Pregnancy".

**Supplementary Information:**

The online version contains supplementary material available at 10.1007/s00592-022-01950-8.

## Introduction

The prevalence of overweight and obesity among women at reproductive age has been increasing in the last years [1, 2]. The 2011 Pregnancy Nutrition Surveillance on maternal health indicators showed a prevalence of 4.5% and 53.7% of women having a pre-pregnancy body mass index (BMI) in the underweight and overweight, respectively [3]. Being overweight or obese increase the risk of adverse pregnancy outcomes (e.g., cesarean delivery, gestational diabetes, gestational hypertension, induction of labor, postpartum hemorrhage, pre-eclampsia) [4]. In addition, the neonates of overweight and obese mothers are at increased risk of admission in the newborn intensive care unit, APGAR scores less than 7 at 5 min, large for gestational age (LGA), macrosomia, compared with mothers with normal BMI [5, 6]. In contrast, the underweight mothers showed increased risk of small for gestational age (SGA) infant and pre-term birth[7].

Gestational Diabetes Mellitus (GDM) represents a health care burden rising as the frequency of obesity increases worldwide [8]. Several studies documented a strong continuous relationship between maternal glucose concentrations and perinatal complications [9, 10]. Moreover, the Hyperglycemia and Adverse Pregnancy Outcome (HAPO) Study demonstrated also that both maternal GDM and obesity are independently associated with adverse pregnancy outcomes and their combination has a greater impact than either one alone [11]. In Italy a recent work has revealed that half of pregnant women affected by GDM were overweight or obese: (27.7% with a BMI 25–30 kg/m_2_ and 20.5% with a BMI > 30 kg/m^2^) [12].

Whereas management of GDM requires strict glucose control, optimal management of maternal obesity has yet to be defined; a prudent diet and moderate exercise are reasonable recommendations for obese pregnant women [11].

Another important determinant for pregnancy and neonatal outcomes is represented by the entity of the weight gain during pregnancy (GWG)[13, 14]. A meta-analysis of more than 1 million pregnant women, showed that 47% had GWG greater than IOM recommendations and 23% had GWG less than IOM recommendations. Furthermore, the meta-analysis confirmed that GWG greater than or less than guideline recommendations, compared with weight gain within recommended levels, was associated with higher risk of adverse maternal and infant outcomes [15]. Pregnant women with an excessive GWG might experience gestational diabetes [16], hypertension [17], delivery complications such as caesarean section [18–21] and high post-partum BMI [22]. Fetal complications associated with excessive GWG are macrosomia and LGA infants [15]; in long term an increased risk for childhood over-weight and obesity [23], morbidity [24], and mortality [25] has been observed. Inadequate GWG, particularly in underweight women, is associated with increased risk for low birthweight or SGA babies [15] and failure to initiate breastfeeding [18, 26].

These observations, all together, highlight the relevance of assessing the management of nutritional therapy of pregnant women and identifying the adequate GWG for each woman. In 1990, recommendations on GWG were issued by the Institute of Medicine (IOM) [27, 28]. In 2009, IOM published a revised version of the guidelines in response to the obesity epidemic [18]]. Based on a comprehensive literature analysis, optimal GWG range was defined according to the three different pre-pregnancy body mass index (BMI) classification criteria (World Health Organization WHO BMI categories [29] and the National Heart, Lung, and Blood Institute) [30].

The report also urges relevant agencies, scientific societies and organizations to adopt these new guidelines providing counseling on diet and physical activity during pregnancy; in order for these recommendations and the GWG targets to be operationalized by pregnant women, there needs to support an adequate clinical attitude to assist women in achieving GWG associated with “favorable pregnancy outcomes” in all pre-pregnancy BMI categories.

Rate of GWG above IOM recommendations was associated with an increased risk of cesarean section and LGA in all BMI categories, whereas a GWG below IOM recommendations was associated with preterm birth in obese women only and with SGA in normal BMI and obese women [31]. However, despite this evidence inadequate knowledge of IOM recommendations and marginal improvement in the amount of GWG have been observed.

Prenatal care providers should know the GWG goals according to IOM recommendations and be clear in their communication about GWG targets to improve peri-natal outcomes, through novel lifestyle interventions in each different prenatal care settings.

Based on this background the Diabetes and Pregnancy Study Group of the Italian Society of Diabetology (SID) and of the Association of Clinical Diabetologists (AMD) designed an ad-hoc questionnaire administered to their Health Professionals members directly involved in the management of nutritional therapy in pregnancy complicated by diabetes.

The survey aims to assess:

1. The knowledge, attitude, and practice of the 2009 IOM recommendations in Italy.
2. The nutritional knowledge and practice of physicians and health professionals about the dietary treatment of pregnant women with diabetes on the national territory.

The results of the survey are reported and discussed as follows.

## Methods

A cross-sectional survey was conducted by using an online self-administered questionnaire. The AMD and the SID invited their members (physicians, dietitians/ nutritionists) managing women with diabetes during pregnancy to complete 15 structured questions survey (Table 1). The invitation to complete the survey was e-mailed to all AMD-SID members (approximately 2400 professionals) two times over a period of 3 months, between October and December 2021.

**Table 1:** Results of the joint Diabetes and Pregnancy Study Group of the AMD and the SID survey on nutrition in pregnancy complicated by diabetes

Part I: General information about the compiler-healthcare professionals	
1. In which Italian region do you predominantly practice in the setting of diabetes?	%
Abruzzo	1.4
Basilicata	0.9
Calabria	3.7
Campania	4.6
Emilia Romagna	11
Friuli Venezia Giulia	6.1
Lazio	11.4
Liguria	2.7
Lombardia	14.6
Marche	1.8
Piemonte	5
Puglia	7.3
Sardegna	5.5
Sicilia	5.9
Toscana	7.3
Trentino Alto Adige	1.8
Umbria	0.9
Valle d’Aosta	0.5
Veneto	7.3
2. Which is your specialty?	
Diabetes	25.6
Endocrinology	53
Internal medicine	10.5
Other (nutrition, diet)	11
3. How long have you obtained your specialty indicated above?	
10–20 years	27.4
5–10 years	13.2
< 5 years	16
> 20 years	42.5
Not specified	0.9
4. Your facility do you have an outpatient clinic with focus on “Diabetes and Pregnancy”?	
Yes	64.8
No	35.2
5. Please indicate, on average, how many women with GDM you follow per year:	
< 10	16.9
10–49	28.8
50–99	17.8
> = 100	36.1
Not specified	0.5
6. Please indicate, on average, how many women with type 1 or type 2 diabetes in pregnancy you follow per year:	
0	2.3
< 5	29.7
5–10	30.6
> 10	36.1
Not specified	1.4
7. How often are prenatal visits for GDM/pre-pregnancy diabetes scheduled?	
Less than once a month	2.3
Once a month	24.7
Every 2 weeks	66.7
More than twice a month	3.2
Not specified	3.2

Part II: Nutrition during pregnancy	
1. How do you determine the patient’s calorie needs at the beginning of the diet treatment? (Multiple answers allowed)	%
According to IOM (Institute of Medicine) recommendations	64.8
According to the dietary history	59.4
According to a written dietary recall	25.8
2. Is the patient weight measured by the health care provider at every prenatal visit?	
Yes, at the first prenatal visit while self-reported weight is collected at every following visits	2.7
Yes, at every visit with the same scale	86.3
Yes, at every visit but with different scales	7.3
No	0.9
Not specified	2.7
3. Do you assess at each prenatal visit if weight gain in the second and third trimester for pre-pregnancy BMI is according to IOM recommendations?	
Yes	97
No	3
If not, what the reason why do not follow the IOM guidelines?	
Lack of dedicated staff	48
Lack of time	36
I don’t follow the IOM guidelines	21
4. Who is responsible for providing dietary information to pregnant women with diabetes?	
Medical doctor	31.1
Dietitian/nutritionist	30.1
Medical doctor and dietitian/nutritionist	38.8
5. Do you have a dietitian/nutritionist in your workplace?	
Yes	60.3
No	39.7
If yes, does the dietitian/nutritionist work in the same facility as you?	
Yes	43.4
Yes, in the same facility but in an outpatient clinic far from mine	7.8
No	9.1
6. Do you usually collect a dietary history to pregnant women before providing nutritional advice?	
Yes, always	81.7
Only the most complicated cases	15.5
No	2.8
7. Do the pregnant women attend to group nutrition education sessions in your workplace?	
Yes	9.6
No	90.4
8. Do you usually provide a diet plan for pregnant women with diabetes?	
Yes	85.4
No, just dietary advice	14.6
If yes, what kind of tools are used?	
General diet meal plan	31.1
Personalized diet based on pregnant women’s needs/preferences	54.3
9. Could you provide women with different needs/preferences with special dietary plans? (Multiple answers allowed)	
No	17.8
Yes, for women with other comorbidities (celiac disease, dyslipidemia, hypertension, …)	74
Yes, vegan/vegetarian women	52.1
Yes, for women from different ethnic groups	57.5
10. Starting the dietary plan do you discuss with the women about the recommended total gestational weight gain?	
Yes	69
No	31
11. What factors should be considered when determining the optimal total gestational weight gain? (Multiple answers allowed)	
Pre-pregnancy BMI	95.9
Current gestational weight gain	79.9
Twin pregnancy	54.0
12. Do you provide stepwise tailored dietary changes at each prenatal visit? (Multiple answers allowed)	
No	10
Yes, in presence of inadequate weight gain (weight loss or excessive weight gain during pregnancy) and/or inadequate fetal growth	78.1
Yes, I provide an increased calorie intake and dietary protein in the transition from the second to the third trimester	53
Yes, in relation to the pattern of food intake/”feel hungry” (calorie re-distribution throughout the day) and physical activity	49.8
13. What is the minimum recommended calorie intake per day for women with gestational diabetes?	
1400	7.8
1500	30.1
1600	62.1
14. What minimum percentage of carbohydrates per day should a woman with gestational diabetes eat?	
30–40	4.1
45–50	73.5
50	20.5
Not known	1.8
15. What should be included in a meal plan for women with diabetes in pregnancy?	
3 main meals	2.7
3 main meals and 1 snack	6.8
3 main meals and 2–3 snacks	89
We do not provide information about the meal plan	1.4

## Results

### Characteristics of survey participants

Two hundred and twenty healthcare professionals completed the survey, overall 109 were anonymous. The sample was representative of the whole national territory (Table 1). The majority of them were physicians (89%), mainly endocrinologists and diabetologists (88%), whereas the remaining 11% of the survey participants were dieticians and nutritionists. Seventy percent of participants had at least 10 years’ experience in treating diabetes in pregnancy and the 65% of the healthcare professionals work in "Diabetes in Pregnancy clinics”. As shown in Table 2, Diabetes Clinics with specific focus on “Diabetes in Pregnancy” provide specialized and personalized care for women with diabetes in pregnancy as compared to sites not focused on this field. Outpatient clinics dedicated to Diabetes in Pregnancy are characterized by: (a) higher number of patients with GDM visited per year (> 100/year: 49% vs. 13%; *p* < 0.0001), (b) higher number of women with pre-pregnancy diabetes followed per year (> 10/year: 46% vs. 18%; *p* < 0.0001), (c) the presence of a dietitian/nutritionist in the clinic (74% vs. 42%; *p* < 0.0001) and d) higher frequency of prenatal visits per month (at least every 15 days 75% vs. 53%; *p* < 0.0001). Moreover, professionals integrated in a team dedicated to "Diabetes in Pregnancy" are usually more accustomed to periodically reviewing the dietary plan and to take into consideration more pointers for nutritional management and the GWG goals (Table 2). However, no differences in term of clinical practice and indications regarding the nutritional program prescriptions (composition of the diet, meals plan) emerged between professionals affiliated to pregnancy dedicated clinic and the other professionals.

**Table 2:** Main differences in Diabetes Clinic according with their focus on “Diabetes and Pregnancy”

	Focus on diabetes and pregnancy		P
	Yes	No	
Women with gestational diabetes (> 100 / year) (%)	49	13	< 0.0001
Women with pre-gestational diabetes (> 10 / year) (%)	46	18	< 0.0001
Presence of dietician/nutritionist in the Diabetes clinic (%)	74	42	< 0.0001
Frequency of women visits at Diabetes Clinic during pregnancy (at least every 15 days) (%)	75	53	< 0.0001
Body weight measurement (%)	100	97	n.s
Evaluation of weekly weight gain (%)	87	83	n.s
Evaluation of goal for maximum weight gain during pregnancy (%)	71	68	n.s
Elements for identify the goal of maximum weight gain: (%) - pre-pregnancy BMI - weight gain already obtained - presence of twin pregnancy	96 85 71	96 70 35	n.s 0.007 < 0.0001
Individualized diet plan (%)	67	67	n.s
Estimated calorie needs at the start of diet treatment based on: (%) IOM guidelines food history food diary	70 64 27	56 52 26	0.037 n.s n.s
Periodic re-evaluation of the diet plan (%)	94	83	0.007
Reasons for re-evaluating the diet plan: (%) unwanted weight changes and/or inadequate fetal growth transition from the second to the third trimester changes in physical activity and/or a”sense of hunger”	86 57 56	64 47 39	< 0.0001 n.s 0.016
Minimum suggested calorie requirement: (%) 1400 kcal 1500 kcal 1600 kcal	14 31 65	4 29 57	n.s
Suggested carbohydrates: (%) 30–40% 45–50% > 50%	4 74 19	4 77 22	n.s
Subdivision of the diet into three main meals and 2–3 snacks(%)	92	83	n.s

### Nutrition management in pregnancy complicated by diabetes

According to survey results, the individualized approach to nutritional counseling in women with pregnancy complicated by diabetes is up to a dietitian/nutritionist in 30% of cases, by physicians only in 31% of cases and mainly (39%) in a coordinated manner by both professional figures particularly in clinics managing a greater number of women with diabetes in pregnancy (73% vs. 50%; *p* = 0.021). Only 10% of the respondents carry out group nutrition classes in pregnancy.

At the first prenatal visit the 53% of the survey participants have provided a personalized diet based on the woman's needs/food preferences, while 32% provided a standard diet plan and the remaining 15% provided dietary advice for diabetes in pregnancy. Eighty percent of professionals have specific diet plans for associated diseases with diabetes such as celiac disease, dyslipidemia, hypertension (74%), or for vegan/vegetarian women (52%) or for women belonging to different ethnic groups (54%).

**Fig. 1:**
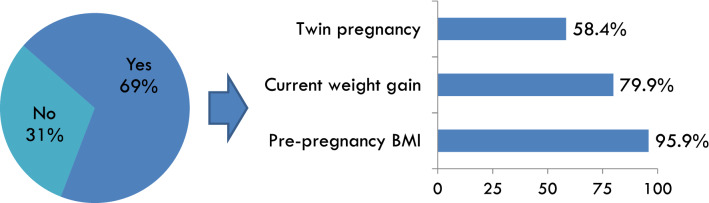
Evaluation of goal for total maximum weight gain during pregnancy and elements taken into consideration to establish the goal

Before providing a dietary plan, 82% of professionals have obtained a dietary history for all the women, whereas the remaining only for the more complex patients. For 97% of women, measured weight was obtained at each prenatal visit (usually with the same scale); whereas in the remaining 3% of women the weight was measured at the first prenatal visit and then the self-reported weight was collected. Pre-pregnancy BMI for GDM women was calculated at the first prenatal visit by self-reported pre-pregnancy weight and measured Hight. For women with pregestational diabetes the pre-pregnancy BMI was recorded in the electronic medical record. In the 87% of women, the GWG was evaluated weekly and compared with IOM guidelines recommendations. When gestational weight gain was not evaluated, the main reasons were: lack of time (36%), lack of dedicated staff (48%) or non-adherence to IOM guidelines (21%). The recommended maximum total weight gain was determined by considering multiple factors including: the pre-pregnancy BMI (96%), the weekly gestational weight gain (80%) and, less frequently, the presence of twin pregnancy (58%) (Fig. 1).

Patient’s calorie needs before nutrition intervention were determined based on IOM recommendations (65%), dietary history (59%) or written dietary recall (26%). The minimum suggested calorie requirement for women with GDM was 1400 kcal for 8%, 1500 kcal for 30% and 1600 kcal for 62% of respondents. The minimum amount (in percent) of suggested carbohydrates was 30–40 for 6%, 45–50 for 74% and > 50 for 21% of respondents. Ninety percent of professionals suggest a meal plan with three main meals and 2–3 snacks.

The 69% of the participants have discussed and shared the recommended total gestational weight gain with the women before providing the dietary plan.

**Fig. 2:**
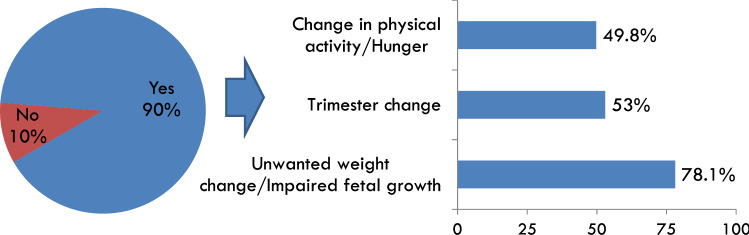
Periodic re-evaluation of the diet plan and reasons for revision

The dietary plan was re-evaluated and/or modified at each scheduled prenatal visit in 90% of cases (Fig. 2). The main reasons behind the modifications of diet plan were: inadequate maternal weight changes (insufficient or excessive weight gain) and/or abnormal fetal growth (78%) and the transition from the second to the third trimester of pregnancy, in relation to the increased energy requirement and to enhance the protein content of the diet (53%), changes in physical activity and/or a “feel hungry” reported by the woman (50%).

## Discussion

This survey found that Italian professionals involved in the management of nutritional therapy are aware of the nutritional needs of women with diabetes in pregnancy. They have a good knowledge of IOM guidelines and they have transferred the recommendations in clinical practice. Nutrition counseling is a cornerstone of prenatal care for all women during pregnancy, specifically in case of GDM and/or obesity [32]. Nutritional status of a woman not only influences her health but also affects pregnancy outcomes and the health of the fetus-neonate [33].

Proper nutrition is fundamental to ensure maternal energy demands and provide to the fetus the essential nutrients for its development and growth. Therefore, it is appropriate to evaluate the energy intake requirement for each pregnant woman. Survey respondents have estimated calorie needs at the beginning of diet treatment mainly based on IOM recommendations and less frequently based on women dietary history or written dietary recall.

Unless for underweight women, during the first trimester, caloric needs should not be increased. From the second trimester, however, professionals involved in pregnant woman management should take into account the progressive increase of calorie requirement due to the pregnancy progression and therefore the need to reviewing the diet plan periodically [34]. Italian professionals usually reevaluate the diet plan in particular for unwanted weight changes (insufficient or excessive weight gain) and/or inadequate fetal growth, while less frequently the transition from the second to the third trimester of pregnancy has been considered. During the transition is essential to increase the calories intakes in relation to the higher energy and protein requirement or for possible changes in physical activity and/or a “feel hungry” reported by the woman.

A correct energy intake should lead to an overall weight gain in line with what is recommended for the different pre-pregnancy BMI classes. Therefore, it is important to evaluate the adequacy of gestational weight gain at each prenatal visit, according to IOM indications (Table 3). It is equally important at first prenatal visit to estimate the total maximum weight gain and to share this information with women. Based on this survey data, Italian professionals are generally compliant with these procedures.

**Table 3:** IOM recommended ranges for total weight gain and for weekly weight gain in the second and third trimester of women, according with pre-pregnancy BMI

	BMI (Kg/m^2^)	Total weight gain range (kg)*	Rates of weight gain kg/week in the 2nd-3rd trimester
Under weight	< 18.5	12.5–18	0.51 (0.44–0.58)
Normal weight	18.5–24.9	11.5–16	0.42 (0.35–0.50)
Over weight	25–29.9	7–11.5	0.28 (0.23–0.33)
Obese	≥ 30	5–9	0.22 (0.17–0.27)

Adapted from Institute of Medicine and National Research Council, 2009 [56]

*Calculations assume a 0.5–2 kg weight gain in the first trimester (based on Siega-Riz et al., 1994; Abrams et al., 1995; Carmichael et al., 1997)

The compliance with the IOM GWG guidelines[35, 36] and the WHO classification appears heterogeneous among Europe and USA. The lack of compliance across countries could be explained by intercountry variability in several factors [37, 38] such as physical activity [39, 40], dietary patterns [40, 41], and psychological or social maternal characteristics [42, 43].

Moreover, some critics emerged regarding the IOM GWG targets which could be overly generous, particularly in class II and III obesity, where a GWG target of 0–4 kg has been recommended [44]. A more restrictive GWG target may be especially important for women with a higher BMI, as end GWG has also been associated with postpartum maternal weight retention and the subsequent risk of becoming overweight [45].

In addition, the UK National Institute for Health and Care Excellence (NICE) did not endorse the IOM recommendations, considering the evidence base insufficient to guide clinical practice (retrospective population-based cohorts) [31].

It must be pointed out that, although IOM recommendations were originally intended for use among American women [18], these recommendations might also be appropriate for women living in western Europe and East Asia [46]. It was also demonstrated by a recent analyses of individual participant data (IPD) from 36 randomized trials (16 countries) that adherence to the IOM recommendations is associated with better pregnancy outcomes [31].

According to our observation, the presence of an outpatient clinic and/or a multidisciplinary team dedicated to "Diabetes in Pregnancy" guarantees a more precise management of women with diabetes in pregnancy and allows to evaluate more specific aspects in the management of dietary therapy and weight gain in pregnancy. All professionals have considered the pre-pregnancy BMI, according with IOM guidelines to determine the goal of total maximum weight gain. However, those professionals working in a dedicated team or in "Diabetes in Pregnancy" clinic have evaluated other determinants such as twin pregnancy or weekly weight gain. This result becomes even more striking considering that an excessive gestational weight gain is already evident at the time of GDM screening (i.e., 24–28 weeks of gestation) in about 15% of the women [47]. Italian data showed that the 2009 IOM recommendations for the gestational weight gain were not respected by almost a half of pregnant women (48%), in particular by overweight or obese women [47], and this is even more evident in England (22%)[48] and in American population (73%) [49], most likely due to differences in psychosocial factors, in diet habits [48] and in obesity prevalence between Europe and USA, and the impact of the ethnic composition of study cohorts. Considering the increasing number of overweight and obese women becoming pregnant, excessive gestational weight gain should be prevented in order to limit the risk of adverse complications to both mother and child. After a diagnosis of GDM, gestational weight gain above the IOM recommendations is associated with increased risks of preeclampsia, requiring hypoglycemic medications, primary cesarean delivery, macrosomia, and LGA without decreasing the odds of SGA or preterm delivery [50]. Moreover, among GDM women, gestational weight gain appears to be a greater risk factor for adverse maternal and fetal outcomes than well treated GDM or pre-pregnancy-BMI [51]. On the other side, inappropriate increase/decrease weight seems to be associated with a significant risk of SGA children[15, 52], a prerequisite for the future development of cardio-metabolic pathologies. Therefore, determining an ideal weight gain after GDM is an important component of healthy pregnancy outcomes in this patient population. Although the total gestational weight gain recommended for overweight or obese women is lower, pregnancy should not be considered a time for weight loss. Therefore, an individualized approach to nutritional counseling needs to take into account the woman’s access to food, the socioeconomic status, race-ethnicity, cultural food choices, but most of all the pre-pregnancy BMI and the presence of twin pregnancy.

To support a full-term pregnancy and not only accounting for increased maternal and fetal metabolism, but also for fetal and placental growth, the caloric intake should increase by approximately 300 kcal/day during pregnancy [53]. This increase should be lower for irrelevant physical activity, whereas in twin pregnancies, the maternal metabolic rate is approximately 10% greater than in singletons. Applying these principles, the caloric requirement could vary in a very broad range. However, the majority of respondents believe that the calorie requirement in pregnancy should not fall below 1600 kcal per day.

Daily energy needs should be obtained by various nutrients. During pregnancy the requirement for proteins increases significantly (especially in the third trimester), while the amount of carbohydrates and fats is unchanged. The protein requirement should provide about 20% of daily energy and a major share of this intake should come from high protein foods biological value or from proteins of animal origin. Carbohydrates are the main source of energy even in pregnancy, their contribution, mainly provided by polysaccharides, must be equal to 45–60% of the energy total daily. In presence of diabetes, the dietary plan should also aim to reduce glycemic fluctuations, especially post-prandial excursions, to avoid ketosis and hypoglycemia in women on drug therapy, and allowing an optimal fetal growth [54].The IOM nutritional guidelines[18] and the Italian Recommended Dietary Allowance[55] advise to guarantee a carbohydrate content of not less than 175 g per day during pregnancy, to allow an adequate substrate for the brain, as this quantity reflects both maternal and fetal cerebral utilization of glucose. Therefore, considering Italian eating habits, national guidelines suggest a carbohydrate content of 40–50% of total energy, preferring foods with low-glycemic index [34]. Italian professionals participating to this survey broadly have agreed with this indication.

## Conclusions

In conclusion, the results of this survey reported the knowledge and attitude of IOM guidelines and the nutritional knowledge and practice of Italian physicians and health professionals about the dietary treatment of diabetes in pregnancy. The application of these recommendations seemed more feasible in clinics/team dedicated to "Diabetes in Pregnancy." It is appropriate an ongoing nutritional education program for the healthcare professionals managing pregnancies complicated by diabetes.

## Supplementary Information

Below is the link to the electronic supplementary material.

Supplementary file1 (ODT 6 kb)
