# Supplementary material for: Knowledge, attitude, and practice of the 2009 Institute of Medicine (IOM) recommendations on the nutritional management of diabetes in pregnancy: an online national survey
Source: Acta Diabetol. 2022 Sep 2;59(12):1597–607. doi: 10.1007/s00592-022-01950-8 (PMC9581811; doi:10.1007/s00592-022-01950-8)
Supplement: Supplementary file 1 [file 592_2022_1950_MOESM1_ESM.odt]

Supplementary materials 

Table S1. Non anonymous participating to the joint Diabetes and Pregnancy Study Group of the AMD and the SID survey on nutrition in pregnancy complicated by diabetes.

| Abruzzo: Gambacorta Francesca. Basilicata: Elefante Antonia. Calabria: Succurro Elena, Sarnelli Paola, Faggiano Francesca, Fava Roberto, De Berardinis Fiorella. Campania: De Chiara Francesco, Saccomanno Franco, Fresa Raffaella, Masulli Maria, Pontillo Alessandro, Fico Francesca. Emilia Romagna: Haddoub Silvia, Magotti Maria Grazia, Monesi Marcello, Agostini Federica, Cavani Rita, Guberti Antonella, Galvan Serena, Taroni Silvia. Friuli Venezia Giulia: Dassi Ferdinando, Li Volsi Patrizia, Velussi Mario, Brunato Barbara, Carpentieri Maria. Lazio: Izzo Valentina, Bitterman Olimpia, Giordani Ilaria, Giordano Laura, Fioriti Elvira, Di Biase Nicolina, Ribaudo Maria Cristina, Buzzetti Raffaella, Mastrilli Valeria, Di Flaviani Alessandra, Abbruzzese Santina, Visalli Natalia, Suraci Concetta, Braucci Sabrina. Liguria: Briatore Lucia, Bruzzone Francesca. Lombardia: Mastropasqua Arturo, Bosi Antonio Carlo, Caretto Amelia, Groppelli Gloria, Ghelardi Renata, Meregalli Giancarla, Scavini Marina, Tresoldi Alberto Stefano, Palmieri Eva, Zerbini Francesca, Gaglio Alessia, Valdes Valeria, Ligari Renato, Gironi Ilaria, Montefusco Laura, Bianchi Elda, Pintaudi Basilio, Beretta Angelo Alessandro, Romano Cristina. Marche: Turchi Federica. Piemonte: Bardinelli Simona, Bonomo Katia, Ponziani Maria Chantal. Puglia: Serra Rosalia, Donvito Rosanna, Cazzetta Giuliana, Laudadio Ester, Braione Antonia Francesca, Dicorato Palma, Fatone Saverio. Sardegna: Clausi Concetta, Meloni Gisella, Casula Stefania. Sicilia: Cascio Marietta, Milluzzo Agostino, Manunta Mario, Greco Domenico, Ridola Giovanni, Pisciotta Maria, Casile Carlo. Toscana: Russo Laura, Malservigi Roberta, de Gennaro Giovanni, Piccini Valentina, Orsini Paola, Ranchelli Anna, Salutini Elisabetta, Dardano Angela, Baggiore Cristina, Ghio Alessandra, Nicolì Francesca, Citro Fabrizia. Trentino Alto Adige: Zambotti Francesca. Umbria: Torlone Elisabetta. Veneto: Lapolla Annunziata, Vinci Carmela, Coracina Anna, Di Sarra Daniela, Morea Aldo Giuseppe, Contin Maria Luisa, Branzi Paola. |
| --- |
